# ﻿Characterisation of the genome and secretome of *Phytophthoracryptogea* and *P.erythroseptica*

**DOI:** 10.3897/imafungus.16.156195

**Published:** 2025-06-10

**Authors:** Yuuri Hirooka, Oscar Villanueva, Ekaterina Ponomareva, Bennett L. Crane, Rick D. Peters, Walid Ellouze, Hai D. T. Nguyen

**Affiliations:** 1 Department of Clinical Plant Science, Faculty of Bioscience and Applied Chemistry, Hosei University, Koganei, 184-8584, Tokyo, Japan Agriculture and Agri-Food Canada, Ottawa Research and Development Centre Ottawa Canada; 2 Agriculture and Agri-Food Canada, Ottawa Research and Development Centre, 960 Carling Avenue, Ottawa, Ontario, K1A 0C6, Canada Hosei University, Koganei Tokyo Japan; 3 Department of Biological Sciences, Brock University, 1812 Sir Isaac Brock Way, St. Catharines, ON L2S 3A1, Canada Brock University St. Catharines Canada; 4 Agriculture and Agri-Food Canada, London Research and Development Centre, 4902 Victoria Avenue North, Vineland Station, ON L0R 2E0, Canada Agriculture and Agri-Food Canada, London Research and Development Centre Vineland Station Canada; 5 Agriculture and Agri-Food Canada, Charlottetown Research and Development Centre, 440 University Avenue, Charlottetown, Prince Edward Island, C1A 4N6, Canada Agriculture and Agri-Food Canada, Charlottetown Research and Development Centre Charlottetown Canada

**Keywords:** Ex-type, Illumina, Nanopore, next generation sequencing, phylogenomics

## Abstract

*Phytophthoracryptogea* Pethybr. & Laff. and *P.erythroseptica* Pethybr. are oomycetes that cause root rot diseases of multiple plant species, including serious diseases of potato. These two species of *Phytophthora* were originally reported in Ireland more than 100 years ago and are closely related phylogenetically and morphologically similar. Both species have wide host ranges and can hybridise with each other. In this study, we sequenced whole genomes of the ex-type strain of *P.cryptogea* (CBS 113.19) and the authentic strain of *P.erythroseptica* (P6180). The genomes of the two isolates were assembled into 52.5 Mb and 74.7 Mb, respectively. A total of 11,654 protein-encoding genes were predicted for *P.cryptogea* and 15,970 for *P.erythroseptica*. Phylogenomic analyses of 2012 single-copy orthologous genes and 99 BUSCO genes from the stramenopiles dataset confirmed that they are sister species and show that they belong to *Phytophthora* clade 8. The secretome analysis of *P.erythroseptica* P6180, *P.cryptogea* CBS 418.71 and *P.cryptogea* CBS 113.19 may provide information for future research on resistance-breeding targets and strategies for pathogen control. This genomic characterisation of the two *Phytophthora* species provides additional reference data that might be useful for future studies on *Phytophthora* genetic variation, pathogenicity and biological traits.

## ﻿Introduction

The genus *Phytophthora* (*Peronosporales*, *Oomycota*) harbours plant pathogenic species that are devastating for agricultural crops, horticultural plants and trees worldwide. The species have previously been characterised by sporangial and antheridial structures, hybridising systems, nutritional strategies and using molecular approaches. Abad et al. (2023a) re-evaluated *Phytophthora* species based on morphological features, phylogenetic analyses and type specimens and 212 species were accepted. Their data are accessible on IDphy: An international online resource for molecular and morphological identification of *Phytophthora* (Abad et al. 2023b). Taxonomically, the genus *Phytophthora* was formerly divided into six groups by morphological and physiological features (Waterhouse 1963; Newhook et al. 1978; Stamps et al. 1990). Förster et al. (2000) studied the phylogenetic relationships amongst *Phytophthora* species using sequence analysis of the ITS1 region. These data demonstrated that *Phytophthora* species can phylogenetically be divided into more groups. Currently, at least ten clades of *Phytophthora* are recognised, based on multi-locus phylogenetic analyses (Kroon et al. 2004; Blair et al. 2008; Martin et al. 2014; Safaiefarahani et al. 2015; Yang et al. 2017; Brasier et al. 2022; Abad et al. 2023a, 2023b).

*Phytophthoracryptogea* Pethybr. & Laff. was described in 1919 as the causal agent of root and stem rot of tomato (*Solanumlycopersicum* L.) in Ireland (Pethybridge and Lafferty 1919). This species is known as a common plant pathogen of herbaceous and woody plants (Delshad et al. 2020; Beal et al. 2021). According to Farr et al. (2025; searched on 05/02/2025), about 380 species have been recorded as host plants of *P.cryptogea* sensu lato. Based on muti-locus phylogenetic analyses, *P.cryptogea* is a member of *Phytophthora* clade 8 and is a sister species of *Phytophthoraerythroseptica* Pethybridge (Kroon et al. 2004; Martin et al. 2014; Safaiefarahani et al. 2015; Yang et al. 2017; Abad et al. 2023a). *Phytophthoraerythroseptica* is well known as a pathogen causing pink rot of potato (*Solanumtuberosum* L.) (Pethybridge 1913). This disease is one of the most damaging diseases of potato and can result in significant economic losses (Platt and Peters 2006). *Phytophthoracryptogea* can also cause pink rot-like symptoms in potatoes, but occurs much less frequently than *P.erythroseptica* in potato tubers (Erwin and Ribeiro 1996). *Phytophthoraerythroseptica* has also been found colonising other host plants in nature (Thapa et al. 2025), while some plant species were reported as hosts for this pathogen after artificial inoculation (Delshad et al. 2020).

According to Abad et al. (2023a), *Phytophthora* clade 8, which currently contains 23 formally described species, is divided into five subclades: clade 8a, 8b, 8c, 8d and 8e. *Phytophthora* clade 8a contains eight species that produce non-papillate sporangia: *P.cryptogea*, *P.drechsleri*, *P.erythroseptica*, *P.kelmanii*, *P.medicaginis*, *P.pseudocryptogea*, *P.sansomeana* and *P.trifolii*, which are considered economically significant pathogens world-wide. Amongst them, *P.cryptogea*, *P.drechsleri*, *P.erythroseptica*, *P.kelmanii* and *P.pseudocryptogea* are proposed to be part of the *P.cryptogea* species complex (Safaiefarahani et al. 2015). The taxonomic status of *P.cryptogea* and *P.erythroseptica* has been under discussion since 1931. Tucker (1931) reported that the two species were similar morphologically, but could be differentiated by their temperature preferences, the size of their oospores, as well as *P.erythroseptica* being a homothallic species. However, subsequent phylogenetic analyses have confirmed the position of *P.erythroseptica* as a distinct species (Kroon et al. 2004; Blair et al. 2008). Interestingly, Mostowfizadeh-Ghalamfarsa et al. (2010) indicated that *P.erythroseptica* isolates may be a secondarily derived homothallic form of *P.cryptogea*, based on morphological, physiological and molecular data. More recently, Safaiefarahani et al. (2015) conducted multi-locus phylogenetic analyses and concluded that *P.cryptogea* and *P.erythroseptica* were distinct species. Although identity of the isolates examined by Safaiefarahani et al. (2015) were confirmed by ITS and β-tubulin sequences obtained from ex-type isolates (CBS 113.19 = P1738 for *P.cryptogea* and CBS 129.23 for *P.erythroseptica*), using type specimens or authentic isolates for additional analyses would provide a more reliable understanding of the taxonomic position of the two species.

Genome-scale phylogeny is a powerful tool to improve our understanding of fungal taxonomy (Li et al. 2021). Currently, genomic data of *P.cryptogea* CBS 418.71 (from the Netherlands, isolated from *Gerbera* sp.) is publicly available, whereas no genome data for any *P.erythroseptica* isolate have yet been reported (Feau et al. 2016). Unfortunately, *P.cryptogea* CBS 418.71 is neither the type nor the authentic culture. An authentic culture is a culture of a species that is used as a reference for taxonomic or comparative purposes verified by a trusted taxonomic expert, usually because the original ex-type culture was lost, no longer viable or hard to access. Comparative secretome analysis of *Phytophthora* species is also an important method for predicting fungal pathogenicity (McGowan and Fitzpatrick 2017). Many types of effector proteins enable pathogens to infect and cause disease in host plants. Some hydrolytic enzymes such as carbohydrate-active enzymes (CAZymes), cutinases, glycoside hydrolases, pectinases and proteases are members of apoplastic effectors and are crucial enzymes for infection of host plants by helping to breach cell walls. Cytoplasmic effectors, another important set of effector proteins, also assist in disease development. For example, the effectors Crinkling and Necrosis (CRN) and RxLR are delivered into plant cells, exploit cellular functions, suppress immune responses and then assist the spread of infection (McGowan and Fitzpatrick 2017). Recently, Villanueva et al. (2024) analysed and detected apoplastic and cytoplasmic effectors of some Canadian *Phytophthoracapsici* isolates and suggested that some might play a role in the isolates’ pathogenicity and adaptability. As mentioned above, *P.cryptogea* and *P.erythroseptica* can infect not only potatoes, but also other plants. Delshad et al. (2020) conducted artificial inoculation assays and demonstrated that the two species can potentially cause disease to many more plant species in nature. The secretome analyses using ex-type or authentic strains might provide important clues about pathogenic evolution of *P.cryptogea* and *P.erythroseptica*, which are closely related.

To address the lack of genomes of ex-type and authentic strains, in this study, we sequenced, assembled and annotated the genomes of the ex-type strain of *P.cryptogea* CBS 113.19 (from Ireland, isolated from tomato or *Petunia*) and an authentic strain of *P.erythroseptica* P6180 (from Ireland, isolated from potato).We then performed phylogenomic analyses to re-evaluate and confirm the taxonomic position of *P.cryptogea* and *P.erythroseptica* in relation to other available *Phytophthora* genomes from mostly ex-type and authentic strains in *Phytophthora* clade 8. Finally, we characterised and compared the secretomes of *P.cryptogea* and *P.erythroseptica*.

## ﻿Methods

### ﻿DNA extraction

The ex-type isolate of *P.cryptogea* CBS 113.19 and authentic strain *P.erythroseptica* P6180 (authenticated by Dr. Gloria Abad of IdPhy: https://idtools.org/phytophthora/) were chosen for sequencing and genome characterisation. The isolates were grown in 2% V8 broth at room temperature and their mycelia were harvested after 10–14 days. DNA was extracted following the protocol of Möller et al. (1992) with some modifications to the tissue lysis step. Harvested mycelia were put into 2-ml screw cap tubes containing 0.5-mm glass beads (Precellys VK05 lysing kit, Bertin, Rockville, Maryland), along with TES buffer (100 mM Tris pH 8.0, 10 mM EDTA, 2% SDS), RNase-A/T1 cocktail (Thermo Fisher Scientific, Waltham, Massachusetts) and proteinase K. Lysis was achieved by shaking tubes in a Precellys24 tissue homogeniser (Bertin) for 40 s at a speed of 6000 rpm. Tubes were incubated at 65 °C for 1 h and subsequent steps were performed following the original protocol. The DNA pellet was then re-suspended in 0.1× TE buffer containing 50 μg/ml RNase A and tubes were incubated at 65 °C for 10 min. Prior to next generation sequencing (NGS), the identity of the isolates was verified by DNA barcode sequencing and analysis of ITS and COX1 following protocols of Robideau et al. (2011) (data not shown).

### ﻿Genome sequencing

Genome sequencing was performed on an Illumina MiSeq instrument and on the Oxford Nanopore MinION platform at the Molecular Technologies Laboratory (MTL), at Ottawa Research and Development Centre (Agriculture and Agri-Food Canada). Briefly, the gDNA of *P.cryptogea* CBS 113.19 and *P.erythroseptica* P6180 were normalised to 400 ng and 350 ng, respectively and were mechanically sheared to 550 bp insert using a Covaris M220 instrument (Covaris, Woburn, Massachusetts). The insert fragments obtained were used as a template to construct PCR-free libraries with NxSeq AmpFREE Low DNA Library kit (Lucigen) according to the manufacturer’s instructions. Single indexed libraries were pooled and paired-end (2 × 300 bp) sequencing was carried out on an Illumina MiSeq instrument. Long read genome sequencing was performed using the Oxford Nanopore MinION system following the manufacturer’s 1D Long fragment protocol. Sequencing ran for 60 and 48 hours, respectively and resulted in N50 of 5.71 Kb for *P.cryptogea* and 25.26 Kb for *P.erythroseptica*.

### ﻿Genome assembly and genome annotation

To assemble the genome of *P.cryptogea* CBS 113.19 and *P.erythroseptica* P6180, raw Nanopore long reads were filtered using Filtlong v.0.2.1 (https://github.com/rrwick/Filtlong), wherein reads shorter than 1 Kb were discarded and the worst reads were removed, until only 6 Gb remained, while keeping the best 90% of reads (options: --min_length 1000 --keep_percent 90 --target_bases 6000000000). The remaining reads were assembled using NextDenovo v.2.5.2 (Hu et al. 2024) with default settings, with an estimated genome size of 60 Mb for both species (correct options: read_cutoff = 1k, genome_size = 60 m, sort_options = -m 20 g -t 15, minimap2_options_raw = -t 8, pa_correction = 3, correction_options = -p 15; assemble options: minimap2_options_cns = -t 8, nextgraph_options = -a 1). Illumina reads were checked with FastQC v.0.12.1 (https://www.bioinformatics.babraham.ac.uk/projects/fastqc/). These reads were trimmed with fastp v.0.23.4 (Chen et al. 2018) (options: --low_complexity_filter --trim_poly_g --trim_poly_x --overrepresentation_analysis --dedup --cut_right 15 --length_required 21). The trimmed reads were mapped to the NextDenovo assembly with BWA v.0.7.18 (Li and Durbin 2009) and errors were corrected with Pilon v.1.24 (Walker et al. 2014) with default settings. Genome assembly statistics were calculated with QUAST v.5.2.0 (Gurevich et al. 2013). An RNA dataset of *P.ramorum* NA1 (NCBI SRA Accession No. SRX4550653, run no. SRR7691076) was downloaded and assembled into transcripts with Trinity v.2.8.5 (Haas et al. 2013) using default settings. Genome assemblies of nine other *Phytophthora* species were obtained from NCBI Genomes (see Suppl. material 1). Genome annotation was performed with Funannotate v.1.8.13 (Palmer and Stallch 2019) using a similar methodology to that described in Villanueva et al. (2024), where repeats were masked with TANTAN v.39 (Frith 2011). The assembled RNA of *P.ramorum* NA1 served as transcript evidence for gene prediction, with the additional options (--min_training_models 50, --busco_db alveolata_stramenophiles, --organism other). To evaluate the completeness of the genome annotation procedure, BUSCO analyses, using the stramenopiles_odb10 database, were conducted with BUSCO v.5.7.1 (Manni et al. 2021) running in protein mode. All genome assembly and genome annotation statistics are summarised in Suppl. material 1.

### ﻿Phylogenomics

To verify the phylogenetic position of *P.cryptogea* and *P.erythroseptica* in *Phytophthora* clade 8 using whole genome data, we performed two phylogenomic analyses with 11 *Phytophthora* genomes mostly of type or authentic isolates (Suppl. material 1). Orthologous group analysis was conducted with OrthoFinder v.2.3.12 (Emms and Kelly 2019) on 11 genomes with default settings. A total of 2012 single-copy genes were found to be present in all 11 genomes. Phylogenomic analysis was performed following a similar methodology from Nguyen et al. (2022). Amino acid sequences were aligned with MUSCLE v.5.1 (Edgar 2022) and automatically trimmed with trimAl v.1.5.0 (Capella-Gutiérrez et al. 2009) using the -automated1 option. Maximum Likelihood trees with fast bootstrapping were calculated with RAxML v.8.2.12 (Stamatakis 2014) (options: -m PROTGAMMAAUTO -x 121 -f a -p 123 -N 100). Using the bipartition trees of individual genes and their respective bootstrapping trees, a multilocus bootstrapping analysis was performed with ASTRAL-III v.5.7.8 (Zhang et al. 2018) to obtain the greedy consensus tree as a cladogram. Trimmed alignment summary statistics were calculated with AMAS v.1.0 (Borowiec 2016). The AfterPhylo.pl v.0.9.1 script (https://github.com/qiyunzhu/AfterPhylo) was used to calculate the average bootstrap support of each tree. The topological distance (RF distance) between each tree and the ASTRAL-III greedy consensus tree was calculated using the ete3 python library (http://etetoolkit.org/documentation/ete-compare/). In addition, phylogenomic analysis was also performed on 99 BUSCO genes obtained from stramenopiles_odb10 dataset, using the method described above for the 2012 single-copy genes. These metrics are summarised in Suppl. materials 2, 3.

### ﻿Effectors and secretome prediction

Secretome analysis was conducted following the approach described by Villanueva et al. (2024). Functional protein analysis was performed on the strains *P.cryptogea* CBS 113.19, *P.cryptogea* CBS 418.71 and *P.erythroseptica* P6180, using InterProScan 5 (Jones et al. 2014), which classifies proteins into families and predicts domains and important sites. Proteins containing Pfam domains linked to pathogenicity were identified as potential effectors, based on the criteria outlined by McGowan and Fitzpatrick (2017). Carbohydrate-active enzymes (CAZymes) were predicted using the dbCAN3 web server (Zheng et al. 2023) for the same strains. Protein sequences were analysed across the following databases: HMMER: dbCAN (E-value < 1e^−15^, coverage > 0.35), DIAMOND: CAZy (E-value < 1e^−102^) and HMMER: dbCAN-sub (E-value < 1e^−15^, coverage > 0.35). Proteins identified as CAZymes were further classified as secreted if a signal peptide was predicted using SignalP 4.1 (Petersen et al. 2011). To identify RxLR and CRN effectors, the *effectR* R package was employed (Tabima and Grünwald 2019). Regular expression-based searches detected motifs characteristic of RxLR (RxLR-EER) and CRN (LFLAK-HVLV) effectors. Gene orthology analysis for RxLR and CRN effectors was performed using OrthoVenn3 (Sun et al. 2023). The OrthoMLC algorithm (Li et al. 2003) was applied with an E-value threshold of 1e^−15^ to identify orthologous gene clusters.

## ﻿Results and discussion

### ﻿Genome sequencing, assembly, and annotation

Nanopore sequencing yielded approximately 16 Gb (3.8 million reads) for *P.cryptogea* CBS 113.19 and 22 Gb (1.5 million reads) for *P.erythroseptica* P6180, while Illumina sequencing yielded approximately 4.2 Gb (15.0 million reads) for *P.cryptogea* CBS 113.19 and 3.7 Gb (13.7 million reads) for *P.erythroseptica* P6180. The assembly and annotation statistics of the three strains of *P.cryptogea* and *P.erythroseptica* are shown in Table 1. *Phytophthoracryptogea* CBS 113.19 had a genome size of 52.5 Mb, assembled into 579 contigs with an N50 of 115.3 Kb. It had 11,654 predicted genes and a GC content of 53.65%, with an 86% BUSCO protein completeness. Compared to the *P.cryptogea* CBS 418.71 genome that was already publicly available, our *P.cryptogea* genome is more contiguous, but had similar genome size. *Phytophthoraerythroseptica* P6180 had a larger genome size at 74.7 Mb and assembled into 51 contigs, with an N50 of 2413.8 Kb. It contained 15,970 predicted genes and achieved a 99% BUSCO protein completeness. We achieved similar or better assembly metrics compared to the existing genome of *P.cryptogea* CBS 418.71.

**Table 1.** Assembly and annotation statistics of *Phytophthoracryptogea* and *P.erythroseptica*.

Species	*P.cryptogea* CBS 113.19	*P.cryptogea* CBS 418.71	*P.erythroseptica* P6180
**Total size (bp) (>= 1000 bp)**	52498254	52430671	74662694
**Number of contigs (>= 1000 bp)**	579	9492	51
**N50 (bp)**	115362	10448	2413823
**L50 (bp)**	122	1322	12
**Longest contig (bp)**	614123	138147	4842162
**GC (%)**	53.65	52.30	52.93
**Coverage (x)**	308	345	295
**BUSCO duplication (stramenopiles) (%)**	0	12	2
**BUSCO protein completeness (stramenopiles)**	86	97	99
**Total number of proteins**	11654	16299	15970
**Secreted proteins**	1520	1822	2162
**GenBank WGS accession**	JBFSGI01	AUWJ02	JBFSGH01

### ﻿Phylogenomics

**Figure 1.**
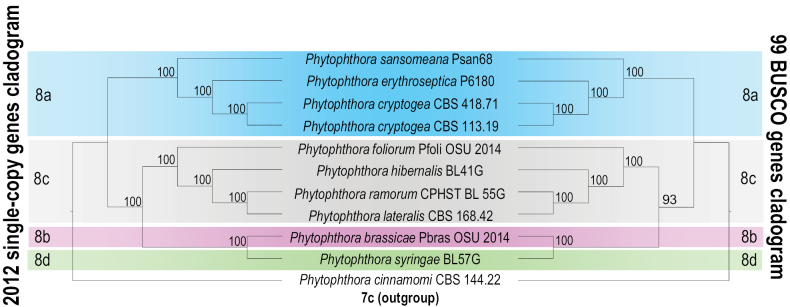
Comparison between the 2012 single-copy genes and 99 BUSCO genes cladograms.

We performed two phylogenomic analyses using: 1) the amino acid sequences of the 2012 single-copy genes found by OrthoFinder and 2) the amino acid sequences of the 99 detected BUSCO genes in the stramenopiles_odb10 dataset. The amino acid trimmed alignments of single-copy genes had an average length of 468 sites where ~ 31% of sites were variable on average, whereas those of the BUSCO genes had an average length of 499 sites where ~ 21% of sites were variable on average. Maximum Likelihood analyses with bootstrapping were performed on each trimmed alignment. The average bootstrap of the maximum likelihood trees generated were 71% and 72% using the alignments of the single-copy genes and BUSCO genes, respectively. To obtain the overall signal and find nodes that represent genealogical concordance, a greedy consensus cladogram was generated using ASTRAL for the single-copy genes and BUSCO genes (Fig. 1). The Maximum Likelihood trees, in both analyses, resembled the final consensus cladogram by 86% on average (see %ref_br in Suppl. materials 2, 3).

Phylogenomics using BUSCO genes is becoming a more common method to understand the relationships between fungi (Li et al. 2021; Manni et al. 2021). When comparing the cladograms generated from the single-copy genes and BUSCO genes, branching patterns and bootstrap support of the two trees were essentially identical (Fig. 1). The bootstrap support was found to be different for only one branch, with 93% in the cladogram generated from the BUSCO genes vs. 100% in the cladogram of single-copy genes. This branching pattern is consistent with previous phylogenetic trees produced by several researchers (Martin et al. 2014; Yang et al. 2017; Burgess et al. 2021; Abad et al. 2023a), although five species of clade 8a, six species of clade 8b and two species of clade 8d are still missing from our analysis. The relationship between *P.cryptogea* and *P.drechsleri*, for instance, has been a matter of controversy, but the genome of *P.drechsleri* is not yet publicly available (Mostowfizadeh-Ghalamfarsa et al. 2010). Sequencing and adding these missing genomes, ideally derived from authentic or type strains, to our phylogenomic analyses would provide more insight into the relationship of species within *Phytophthora* clade 8.

The gene OG0006696, one of the 2012 single-copy genes, could be a useful barcode gene for *Phytophthora* clade 8 because this gene was detected in all the 11 genomes analysed, the average bootstrap branch support was 98% and the topology was 100% identical to the greedy consensus cladogram. The OG0006696 gene encodes the NUP96 protein that is a key component of the nuclear pore complex as determined by InterProScan 5 with Pfam database. This is responsible for regulating the transport of molecules between the nucleus and the cytoplasm in eukaryotic cells (Beck and Hurt 2017).

### ﻿Secretome variation and functional classification

The predicted secreted proteins were categorised as apoplastic or cytoplasmic and grouped by function, including microbe-associated molecular patterns (MAMPs), CAZymes, protease inhibitors, necrosis- and ethylene-inducing peptide 1-like proteins (NLPs), cutinases and key effectors such as RxLR and CRN (Table 2, Suppl. materials 4, 5). Secretome comparisons amongst *P.erythroseptica* P6180, *P.cryptogea* CBS 418.71 and *P.cryptogea* CBS 113.19 reveal marked differences.

**Table 2.** Total number of predicted effector gene candidates in *Phytophthoracryptogea* CBS 113.19, *P.cryptogea* CBS 418.71 and *P.erythroseptica* P6180 strains. The numbers represent genes encoding predicted secreted proteins.

Category		Family	Number of proteins per strain		
			*P.cryptogea* CBS 113.19	*P.cryptogea* CBS 418.71	*P.erythroseptica* P6180
**MAMP**		Sterol-binding proteins	168	126	207
		Transglutaminase proteins	16	28	21
**Apoplastic effectors**	CAZymes	Total	833	1005	1048
		Secreted	193	202	278
	Protease/inhibitor	Glucanase	49	90	70
		Kazal	51	100	98
		Cathepsin	6	10	8
		Cystatin	2	2	4
	Others	Necrose-inducing proteins (NLPs)	39	92	120
		Cutinases	4	6	7
**Cytoplasmic effectors**	RxLR	RxLR and EER motifs (complete)	78	105	149
		RxLR motif only	6	6	12
		EER motif only	41	11	32
		No motifs	22	17	72
	CRN	HVLV and LFLAK motifs (complete)	1	3	6
		HVLV motif only	5	10	25
		LFLAK motif only	6	9	5
		No motifs	10	21	16

*Phytophthoraerythroseptica* P6180 had the highest number of secreted sterol-binding proteins (207), followed by *P.cryptogea* CBS 113.19 (168) and *P.cryptogea* CBS 418.71 (126). Secreted transglutaminases were most abundant in *P.cryptogea* CBS 418.71 (28), compared to *P.erythroseptica* P6180 (21) and *P.cryptogea* CBS 113.19 (16). CAZyme-encoding genes varied across strains. *Phytophthoraerythroseptica* P6180 had the highest totals, with 1,048 genes overall and 278 secreted, followed by *P.cryptogea* CBS 418.71 (1,005 total, 202 secreted) and *P.cryptogea* CBS 113.19 (833 total, 193 secreted). Protease inhibitor counts were highest in *P.cryptogea* CBS 418.71 (202), followed by *P.erythroseptica* P6180 (180) and *P.cryptogea* CBS 113.19 (108). *Phytophthoracryptogea* CBS 418.71 exhibited the highest counts of glucanase, kazal and cathepsin, with 90, 100 and 10 predicted proteins, respectively. This was followed by *P.erythroseptica* P6180, which had 70 glucanase, 98 kazal and 8 cathepsin predicted proteins. *Phytophthoracryptogea* CBS 113.19 had the lowest counts, with 49, 51 and six predicted proteins for glucanase, kazal and cathepsin, respectively. For cystatin, *P.erythroseptica* P6180 had the highest count, with four predicted proteins, while *P.cryptogea* CBS 113.19 and *P.cryptogea* CBS 418.71 had two predicted proteins each. NLPs were most abundant in *P.erythroseptica* P6180 (120), compared to *P.cryptogea* CBS 418.71 (92) and *P.cryptogea* CBS 113.19 (39). Cutinases, which enable pathogens to penetrate epidermal plant defences, were most numerous in *P.erythroseptica* P6180 (7), followed by *P.cryptogea* CBS 418.71 (6) and *P.cryptogea* CBS 113.19 (4).

Concerning CRN-predicted effectors, *P.erythroseptica* P6180 contained six proteins with complete motifs, 16 without motifs, 25 with only the HVLV motif and five with only the LFLAK motif. *Phytophthoracryptogea* CBS 418.71 displayed the highest number of proteins lacking motifs (21) and included three proteins with complete motifs, 10 with HVLV motifs and nine with LFLAK motifs. Meanwhile, *P.cryptogea* CBS 113.19 contained one protein with complete motifs, five with HVLV motifs, six with LFLAK motifs and 10 proteins without motifs. Orthology cluster analysis of CRN-predicted effectors identified 10 shared clusters amongst all strains, with two unique clusters in *P.erythroseptica* P6180, one in *P.cryptogea* CBS 418.71 and one in CBS 113.19 (Suppl. material 4).

For RxLR-predicted effectors (Table 2), *P.erythroseptica* P6180 contained 149 complete effectors, 72 without motifs, 12 with only the RxLR motif and 32 with only the EER motif. In comparison, *P.cryptogea* CBS 113.19 included 78 complete effectors, 22 without motifs, six with only the RxLR motif and 41 with only the EER motif, while CBS 418.71 had 105 complete effectors, 17 without motifs, six with only the RxLR motif and 11 with only the EER motif. Orthology cluster analysis of RxLR-predicted effectors identified 31 shared clusters amongst all strains, with 12 unique clusters in *P.erythroseptica* P6180, two in *P.cryptogea* CBS 418.71 and four in CBS 113.19 (Suppl. material 5).

## ﻿Conclusion

We characterised the genomes of type and authentic isolates of *P.cryptogea* and *P.erythroseptica*, adding to the publicly available genomic resources of *Phytophthora* for future studies. Phylogenomic analyses confirmed the evolutionary and taxonomic relationship between these two species. Historically, hybridisation has been investigated and reported in *Phytophthora* as a common event in their evolution (e.g. Hurtado-Gonzales et al. (2009); Nirenberg et al. (2009); Man in ’t Veld et al. (2012); Bertier et al. (2013); Nagel et al. (2013); Husson et al. (2015); Safaiefarahani et al. (2016)). Genome sequences generated here from authentic isolates of both species extend the reference dataset needed to test hypotheses on hybrid origin and host‑range evolution, while our analysis of effectors might be useful for future studies on targets for resistance breeding and pathogen management strategies.

## ﻿Additional information

### Adherence to national and international regulations

All the fungal strains used in this study have been legally obtained, respecting the Convention on Biological Diversity (Rio Convention).

### Author ORCIDs

Yuuri Hirooka https://orcid.org/0000-0003-2647-8873

Bennett L. Crane https://orcid.org/0009-0004-8667-6200

Walid Ellouze https://orcid.org/0000-0002-8749-9486

### Data availability

Raw sequence data and genome assemblies were uploaded to NCBI under BioProject PRJNA1140244. Genome annotation, phylogenomics and effectors analysis files are available for download on Zenodo (10.5281/zenodo.14919761). The raw data of Nanopore and Illumina se-quencings were deposited in NCBI SRA under study SRP568312.

## Supplementary materials

Supplementary material 1 Genome statistics of *Phytophthora* genomes in the phylogenomic analysis Data type xlsx https://binary.pensoft.net/file/1349299This dataset is made available under the Open Database License (http://opendatacommons.org/licenses/odbl/1.0/). The Open Database License (ODbL) is a license agreement intended to allow users to freely share, modify, and use this Dataset while maintaining this same freedom for others, provided that the original source and author(s) are credited.Yuuri Hirooka, Oscar Villanueva, Ekaterina Ponomareva, Bennett L. Crane, Rick D. Peters, Walid Ellouze, Hai D. T. Nguyen

Supplementary material 2 2012 single copy genes statistics Data type xlsx https://binary.pensoft.net/file/1349300This dataset is made available under the Open Database License (http://opendatacommons.org/licenses/odbl/1.0/). The Open Database License (ODbL) is a license agreement intended to allow users to freely share, modify, and use this Dataset while maintaining this same freedom for others, provided that the original source and author(s) are credited.Yuuri Hirooka, Oscar Villanueva, Ekaterina Ponomareva, Bennett L. Crane, Rick D. Peters, Walid Ellouze, Hai D. T. Nguyen

Supplementary material 3 99 BUSCO genes statistics Data type xlsx https://binary.pensoft.net/file/1349301This dataset is made available under the Open Database License (http://opendatacommons.org/licenses/odbl/1.0/). The Open Database License (ODbL) is a license agreement intended to allow users to freely share, modify, and use this Dataset while maintaining this same freedom for others, provided that the original source and author(s) are credited.Yuuri Hirooka, Oscar Villanueva, Ekaterina Ponomareva, Bennett L. Crane, Rick D. Peters, Walid Ellouze, Hai D. T. Nguyen

Supplementary material 4 Venn diagram depicting the number of shared CRN-predicted protein clusters among the various *Phytophthora* strains Data type pptx https://binary.pensoft.net/file/1349302This dataset is made available under the Open Database License (http://opendatacommons.org/licenses/odbl/1.0/). The Open Database License (ODbL) is a license agreement intended to allow users to freely share, modify, and use this Dataset while maintaining this same freedom for others, provided that the original source and author(s) are credited.Yuuri Hirooka, Oscar Villanueva, Ekaterina Ponomareva, Bennett L. Crane, Rick D. Peters, Walid Ellouze, Hai D. T. Nguyen

Supplementary material 5 Venn diagram showing the number of shared RxLR-predicted protein clusters among the various *Phytophthora* strains Data type pptx https://binary.pensoft.net/file/1349303This dataset is made available under the Open Database License (http://opendatacommons.org/licenses/odbl/1.0/). The Open Database License (ODbL) is a license agreement intended to allow users to freely share, modify, and use this Dataset while maintaining this same freedom for others, provided that the original source and author(s) are credited.Yuuri Hirooka, Oscar Villanueva, Ekaterina Ponomareva, Bennett L. Crane, Rick D. Peters, Walid Ellouze, Hai D. T. Nguyen
