# Supplementary material for: ﻿Characterisation of the genome and secretome of Phytophthoracryptogea and P.erythroseptica
Source: IMA Fungus. 2025 Jun 10;16:e156195. doi: 10.3897/imafungus.16.156195 (PMC12177512; doi:10.3897/imafungus.16.156195)
Supplement: Supplementary material 4 — Venn diagram depicting the number of shared CRN-predicted protein clusters among the various Phytophthora strains [file imafungus-16-e156195-s004.pptx]

## Slide 1
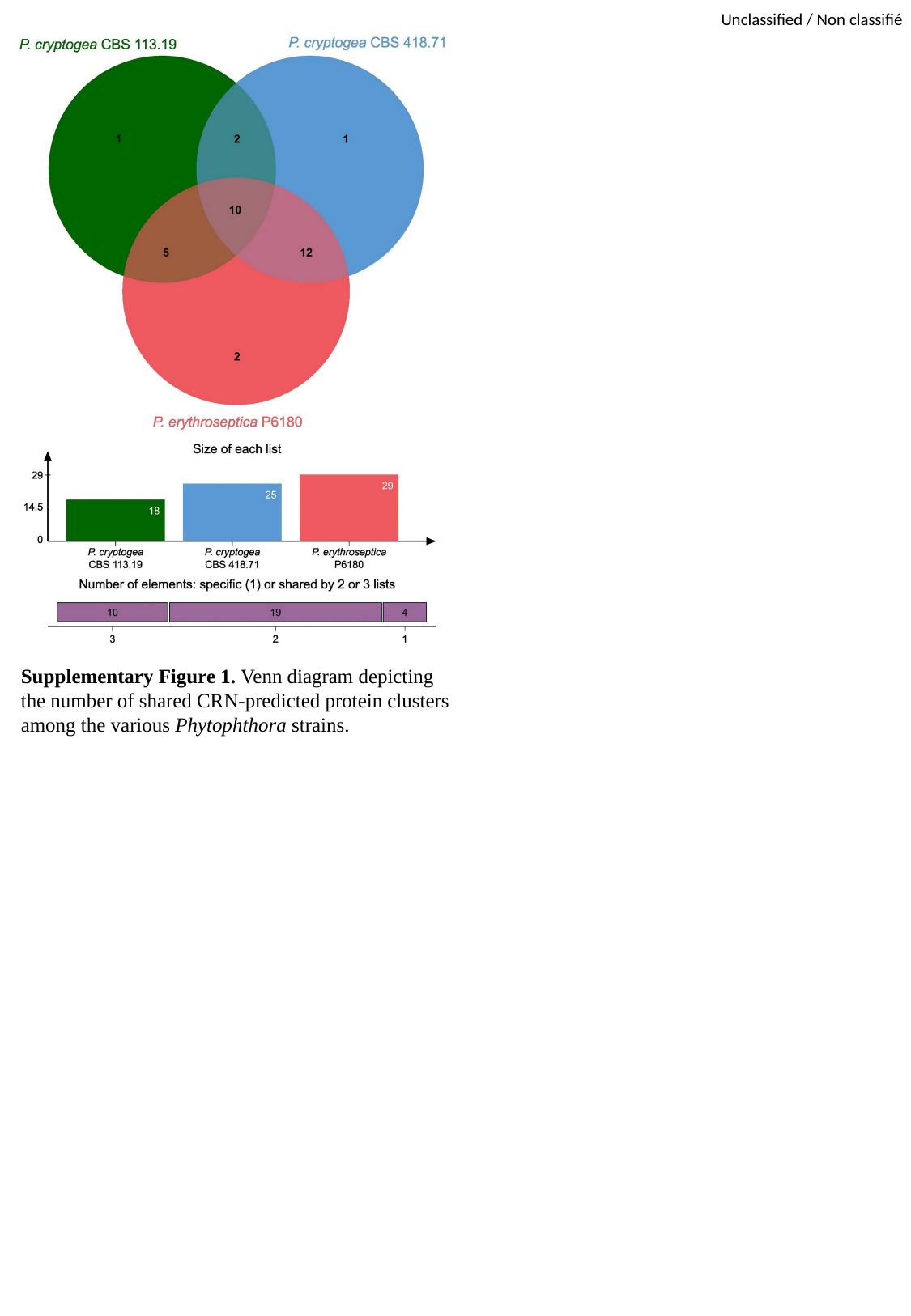

Supplementary Figure 1. Venn diagram depicting the number of shared CRN-predicted protein clusters among the various Phytophthora strains.

## Slide 2
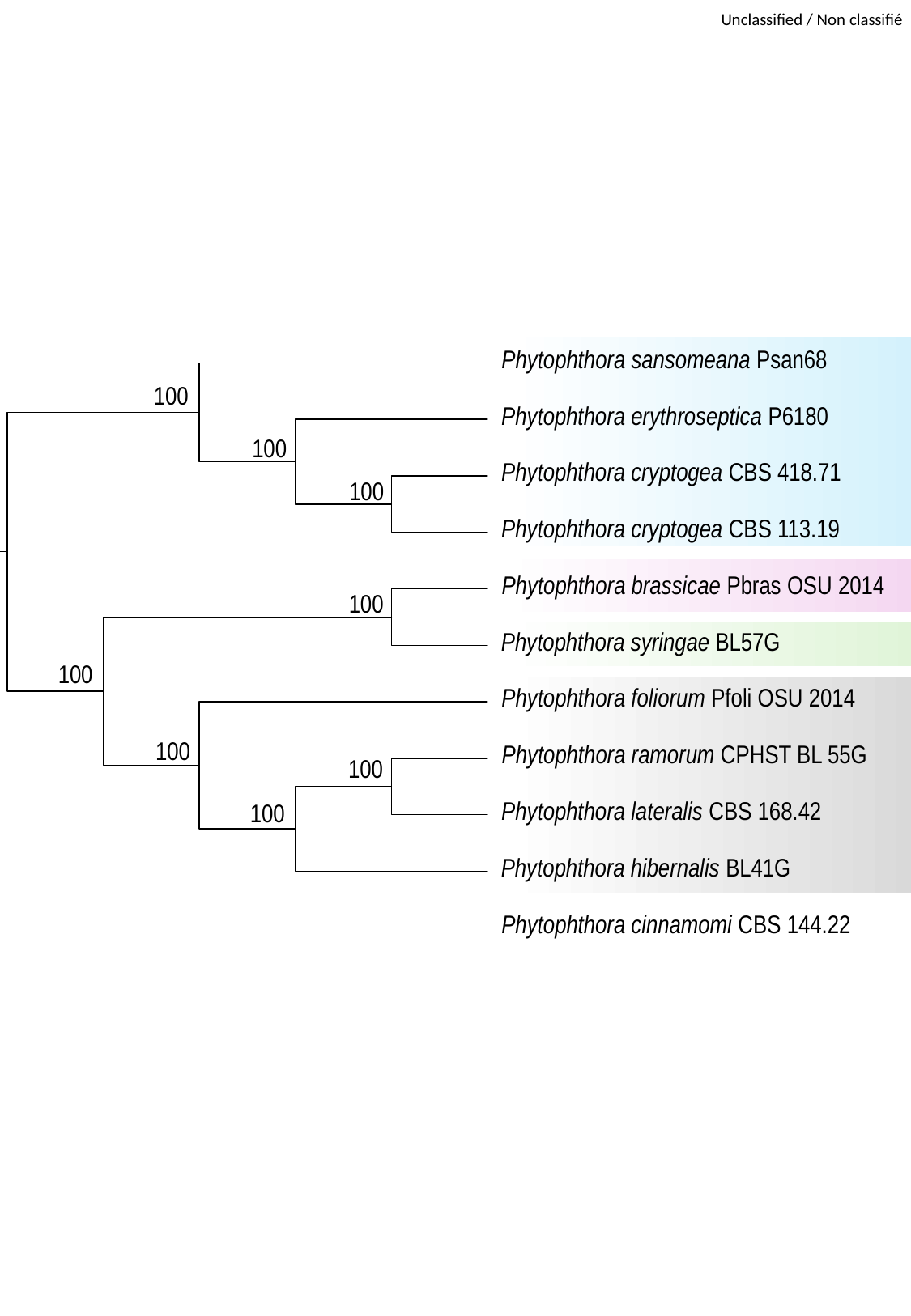

Phytophthora sansomeana Psan68
100
Phytophthora erythroseptica P6180
8a
100
Phytophthora cryptogea CBS 418.71
100
Phytophthora cryptogea CBS 113.19
8b
Phytophthora brassicae Pbras OSU 2014
100
8d
Phytophthora syringae BL57G
100
Phytophthora foliorum Pfoli OSU 2014
100
Phytophthora ramorum CPHST BL 55G
100
8c
Phytophthora lateralis CBS 168.42
100
Phytophthora hibernalis BL41G
7c
Phytophthora cinnamomi CBS 144.22
